# A Randomized Controlled Trial Evaluating Postoperative Port Site Infections Among Patients Undergoing Laparoscopic Cholecystectomy Either via Umbilical or Epigastric Port

**DOI:** 10.7759/cureus.48709

**Published:** 2023-11-12

**Authors:** Ayush Raj, Sakshi Singh, Ankit Raj, Yasir Tajdar

**Affiliations:** 1 Department of General Surgery, Indira Gandhi Institute of Medical Sciences, Patna, IND; 2 Department of Surgery, Indira Gandhi Institute of Medical Sciences, Patna, IND

**Keywords:** pain assessment, epigastric port, umbilical port, gallbladder removal, surgical site infection, laparoscopic cholecystectomy

## Abstract

Background and Objectives: Laparoscopic cholecystectomy (LC) is a keyhole surgical procedure considered a gold standard treatment for benign gallbladder (GB) diseases. GB retrieval is done per the surgeon’s choice through an umbilical or epigastric port. However, postoperative port site infection (PSI) and pain were major complications of this technique. The study aimed to compare the postoperative PSI between epigastric and umbilical ports among patients undergoing LC.

Methods: A prospective randomized controlled trial was conducted among 50 patients who underwent LC for benign GB disease at the Indira Gandhi Institute of Medical Sciences (IGIMS), Patna, for 6 months. Participants were randomized into epigastric port (n=25) and umbilical port (n=25). Postoperatively, PSI on a postoperative day (POD) of 10 and 30, retrieval difficulty score, Postoperative pain (POP) using a visual analog scale (VAS), and port site scar appearance after 6 months were assessed.

Results: This study divided 50 LC patients into epigastric and umbilical ports (n=25). Among them, 31 were females (62%), 19 males (38%), and mean ages of 43.5 ± 10.7 and 40.7 ± 12.6 years were observed for the epigastric and umbilical ports; group age was similar (p=0.37). The gender distribution was similar between groups (p=0.9 for males, p=0.7 for females). The epigastric port displayed a mean body mass index (BMI) of 22.3 ± 1.01, while the umbilical port had a significantly higher mean BMI of 23.7 ± 1.10 (p=0.04). Patients with symptomatic cholelithiasis as the primary reason for surgery were common in both groups (p=0.2 for GB stones, p=0.4 for GB polyps). The mean hospital stays and surgical duration were similar (p=0.7 and 0.99). Epigastric ports had 8% postoperative PSI on POD 10 (vs. 12%, p=0.07) and 0% on POD 30 (vs. 4%, p=1.0), compared to umbilical ports. Umbilical port patients were more satisfied with scar appearance (92% vs. 76%, p=0.11) and less dissatisfied (8% vs. 24%, p=0.02) 6 months post-surgery. Compared to the umbilical port, patients with epigastric ports had significantly higher VAS pain scores at multiple postoperative time points (p-values <0.001 to 0.03). It was also harder to retrieve epigastric port GB (p=0.01).

Conclusion: The current study highlights the importance of port site selection among patients who underwent LC, as it can notably impact postoperative outcomes. While the umbilical port may be associated with lower PSI rates and better cosmetic outcomes, GB retrieval through the epigastric port may result in lower postoperative port site pain. Surgeons should carefully consider these factors when choosing the port site for LC procedures. Further research, including larger multicenter trials, is needed to validate and expand upon these results, ultimately enhancing patient care in GB surgery.

## Introduction

Benign gallbladder (GB) diseases, such as GB stones or polyps, represent a substantial portion of cases contributing to abdominal morbidity [1]. Notably, a significant percentage of individuals, estimated between 50% and 80%, harbor these conditions without experiencing overt symptoms. In comparison, approximately 30% of cases eventually progress to symptomatic states requiring surgical intervention [2,3]. Cholecystectomy, the surgical removal of the GB, stands out as the most prevalent and effective treatment option for individuals grappling with symptomatic conditions [4]. Among the various surgical approaches, laparoscopic cholecystectomy (LC) has emerged as the benchmark, surpassing traditional open techniques, particularly in addressing calculous cholecystitis [5]. This innovative surgical procedure has wrought a revolution in modern surgery, offering an array of advantages that have redefined patient care.

LC, a minimally invasive surgical procedure is the gold standard over traditional open technique in the treatment of calculous cholecystitis [5]. This procedure has revolutionized the field of modern surgery with its advantages of less postoperative pain (POP), superior aesthetic outcomes, and improved patient comfort and satisfaction. Furthermore, this technique has been associated with shorter hospital stays, allowing patients to return to their daily lives more swiftly, and it has proven to be a cost-effective approach, making it accessible to a broader range of patients [5,6].

However, this surgical technique, while offering numerous benefits, is not without its potential complications. One notable complication that has garnered significant attention in clinical practice and research is postoperative port site infection (PSI). Despite the continuous advancements in surgical techniques and the rigorous implementation of infection control measures, PSI remains a persistent challenge that can significantly impact patient recovery and healthcare costs [5]. In the context of LC, the GB is typically removed through either the epigastric or the umbilical port. As this procedure has gained immense popularity over the years, various studies have been conducted to compare the complications associated with epigastric and umbilical ports for GB retrieval [7-10]. These studies aim to shed light on critical aspects of patient care and surgical outcomes. However, it is worth noting that regardless of the acceptance and admiration of research on this technique, there still exists a gap in the data pertaining to the superiority of one port over another in terms of POP and PSI. Hence, this clinical trial was conducted with the objectives to compare the postoperative PSI and to provide a comprehensive understanding of the clinical outcomes associated with the LC using epigastric and umbilical ports.

We hypothesize that there will be no significant difference in POP and PSI rates between these port sites during GB removal in LC. Our findings may influence clinical decisions and patient care in LC, improving outcomes and healthcare practices in GB surgical procedures.

## Materials and methods

Ethical consideration and study design

A parallel, double-arm, prospective randomized controlled trial was conducted in the Department of Surgery at Indira Gandhi Institute of Medical Sciences (IGIMS), Patna, for 6 months. Before the study began, the Institutional Ethics Committee (IEC Number: 562/IEC/IGIMS/2021) granted ethical approval, and written informed consent was obtained from all the participants. The current trial followed the consolidated reporting trial standards (CONSORT) guidelines [11].

Sample size estimation

The sample size was determined using G*POWER Software Version 3.1.9.4, employing a t-test for two independent groups assuming a statistical power of 95% and a significance level of 5%. The effect size (Cohen’s d) was calculated based on the means of postoperative visual analog scale (VAS) scores at 24 hours for the umbilical and epigastric ports, which were 3.05 and 2.15, respectively, with SD of 0.87 for both [7]. With these parameters, the required total sample size was computed as 44. A 10% attrition rate was considered to account for potential attrition, resulting in a rounded sample size of 25 participants per port and a total sample size of 50.

Participants eligibility

All eligible patients aged 17-80 years, admitted to the department for elective LC due to benign GB disease (including symptomatic GB stones or polyps) were considered under the inclusion criteria for the study. However, patients with suspected/confirmed GB malignancy, bleeding disorders, and obstructive jaundice were excluded from the study.

Randomization

Study participants were randomized in a 1:1 ratio using a lottery method to determine whether they would undergo GB removal through the epigastric or umbilical ports. Allocation concealment was ensured by the principal investigator using the serially numbered opaque sealed envelope (SNOSE) technique. This trial was a double-blinded study, meaning that both the participants and the statistician needed to be made aware of which port site intervention was used.

Outcome measures

Outcome measures were collected using a pre-designed and validated tool to record sociodemographic details and clinicopathological measures postoperatively. The primary outcome measure was the incidence of postoperative PSI at the epigastric and umbilical port sites on postoperative days (POD) 10 and 30. Secondary outcomes included the GB retrieval difficulty score, assessed immediately after surgery by the operating surgeon on a scale from 1 (easiest) to 10 (most difficult), and POP at 1, 6, 12, 24, and 36 hours using the VAS, ranging from 0 to 10 for both port sites. VAS scores were assessed by another surgeon unaware of the retrieval site to prevent information bias. Port site scarring was also evaluated 6 months after surgery, with patients providing feedback on their satisfaction with the cosmetic appearance of the scar.

Intervention

**Figure 1:**
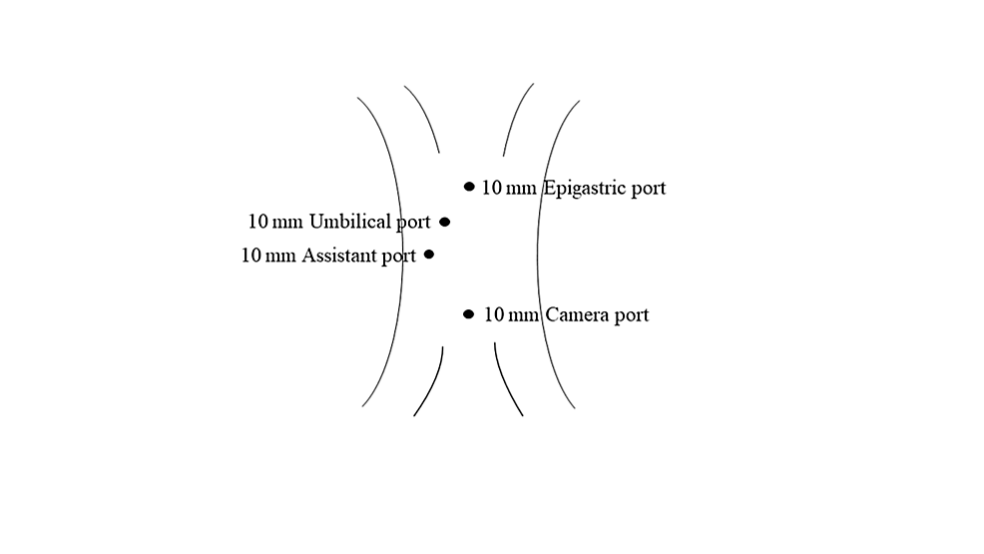
Representation of different port sites for laparoscopic cholecystectomy

All surgeries were performed by consultant general surgeons (having experience of more than 2 years) under general anesthesia. Patients in both groups received preoperative preparation and pre-anesthetic medications and were admitted one day before the surgery. A usual four-port technique was utilized (Figure 1).

The GB was then removed from all patients according to their group allocation using a retrieval bag. Closure of the umbilical and epigastric port fascia and skin was achieved using absorbable vicryl sutures, followed by dressing.

In the initial 24 hours following surgery, postoperative analgesia with pethidine at 0.5 mg/kg was administered intramuscularly every 6 hours. However, orals have been started after 6 hours, and a normal diet after 24 hours after the surgery. Once the diet was initiated, patients received oral analgesics, including paracetamol (1000 mg every six hours) or diclofenac (50 mg every 12 hours). For patients experiencing significant pain (VAS score exceeding 7), additional analgesics such as diclofenac (1.5 mg/kg) and tramadol (1 mg/kg) were administered after every 8 hours.

Statistical analysis

The data were anonymized, coded, and inputted into an Excel worksheet (MS Office, Excel 2016) for subsequent statistical analysis using SPSS Version 26.0 (IBM Corp, Armonk, New York, USA). The Kolmogorov-Smirnov test assessed data normality, indicating that the data followed a normal distribution. Descriptive analyses, including frequency, percentage, mean, SD, and range, were used to present the data. The chi-square test was applied for categorical data, while independent t-tests and ANOVA were used for quantitative data. Statistical significance was determined at a 5% significance level with a 95% confidence level (p<0.05).

## Results

Fifty participants underwent LC and were divided into the epigastric port (n=25) and the umbilical port (n=25) groups. Among these participants, 19 males (38%) and 31 females (62%) were in the study population. Our study compared demographic and clinicopathological factors between patients who had LC through the epigastric port site and those who had it through the umbilical port site (Table 1). Most of the sample were 28-57 years old. The mean age for the epigastric port was 43.5 ± 10.7 years, while the umbilical port had a slightly lower mean age of 40.7 ± 12.6 years, exhibiting no statistically significant difference between the two groups (p=0.37). Regarding gender distribution, the epigastric port comprised 10 male and 15 female participants, while the umbilical port had nine males and 16 females (Table 1). The gender distribution exhibited insignificant statistical variation between the groups (p=0.9 for males, p=0.7 for females). However, a notable distinction emerged when we examined the body mass index (BMI). The epigastric port had a mean BMI of 22.3 ± 1.01, whereas the umbilical port had a higher mean BMI of 23.7 ± 1.10 and the difference was found to be statistically significant (p=0.04; Table 1).

**Figure 2:**
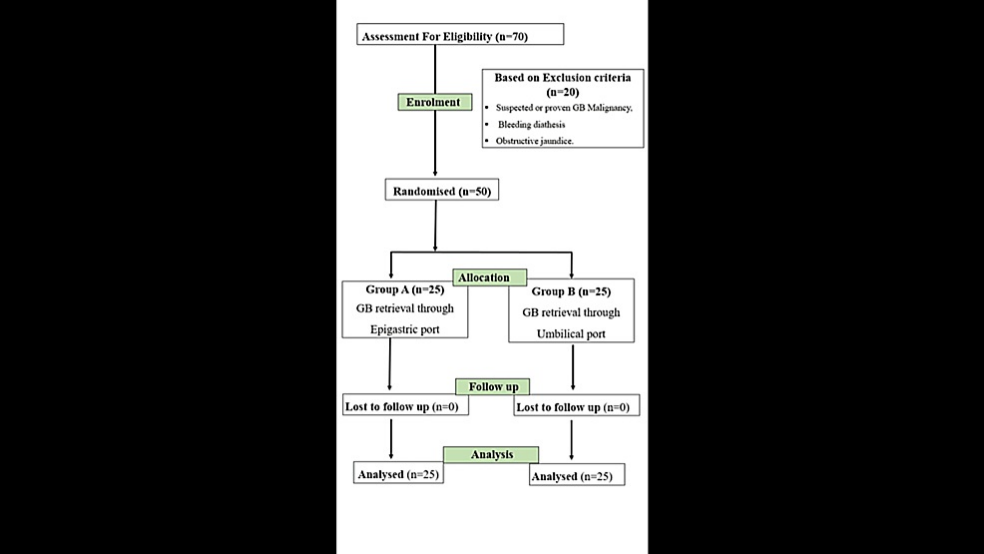
Consolidated reporting trial standards (CONSORT) flowchart illustrating participant enrollment and progression through the study

**Table 1:** Comparison of demographic and clinicopathological variables between epigastric and umbilical port sites in laparoscopic cholecystectomy patients. BMI: body mass index; GB: gallbladder Significant = p<0.05

Baseline variables	Epigastric port (n=25)	Umbilical port (n=25)	p-value
Age	43.5 ± 10.7	40.7 ± 12.6	0.37
Sex			
Male	10	9	0.9
Female	15	16	0.7
BMI	22.3 ± 1.01	23.7 ± 1.10	0.04
Indication for surgery			
Symptomatic GB stones	18	15	0.2
GB polyp	7	10	0.4
Duration of surgery (min)	53.2 ± 10.5	58.9 ± 18.7	0.7
Hospital stay duration (hour)	48 ± 2	50 ± 3	0.99

Among the epigastric port group, 18 participants had symptomatic GB stones, and seven had GB polyps (Table 1). In contrast, the umbilical port consisted of 15 participants with symptomatic GB stones and 10 with GB polyps. However, this finding was found to be statistically insignificant between the two groups (p=0.2 for symptomatic GB stones, p=0.4 for GB polyps; Table 1). When assessing the duration of surgery, we found that the mean duration for the epigastric port was 53.2 ± 10.5 min, whereas the umbilical port had a mean duration of 58.9 ± 18.7 min. This difference in surgical duration did not reach statistical significance (p=0.7; Table 1). Finally, we examined the mean duration of hospital stays, which was 48 ± 2 hours for the epigastric port and 50 ± 3 hours for the umbilical port which exhibits no statistically significant difference in hospital stay duration among the two groups (p=0.99; Table 1).

Table 2 compares postoperative PSI between the epigastric and umbilical ports (Table 2). The study included 25 patients in each port (n=25). On POD 10, the epigastric port had two cases (8%) of PSI, while the umbilical port had three cases (12%). This difference was statistically non-significant, with a p-value of 0.07 (Table 2). At a later follow-up on POD 30, the epigastric port had no reported cases of PSI, whereas the umbilical port had one case (4%). This difference was also statistically non-significant, with a p-value of 1.0. Regarding the cosmetic appearance of port site scarring after 6 months, 19 patients (76%) in the epigastric port expressed satisfaction, while six patients (24%) reported being unsatisfied (Table 2). In comparison, the umbilical port had a higher satisfaction rate, with 23 patients (92%) satisfied and only two (8%) unsatisfied. This difference in patient satisfaction was statistically non-significant, with p-values of 0.11 for satisfied patients and a statistically significant value of 0.02 for unsatisfied patients (Table 2).

**Table 2:** Comparison of postoperative port site infection (PSI) and cosmetic outcomes between epigastric and umbilical port sites in laparoscopic cholecystectomy patients. POD: postoperative day significant = p<0.05

	Epigastric port (n=25)	Umbilical port (n=25)	p-value
PSI			
POD 10	2	3	0.07
POD 30	0	1	1.0
Cosmetic appearance of port site scaring after 6 months			
Satisfied	19	23	0.11
Unsatisfied	6	2	0.02

Our study compared the after-surgery experiences of people with LC through the epigastric and umbilical port sites (Table 3). At the 1-hour post-surgery mark, patients in the epigastric port reported statistically significant higher VAS scores for pain (6.9 ± 1.2) compared to those in the umbilical port (5.1 ± 1.5), with a p-value of 0.001. This trend continued at the 6-hour post-surgery point (epigastric port VAS scores =4.9 ± 0.4; umbilical port VAS score 2.5 ± 1.5), at 12-hour post-surgery interval (epigastric port VAS scores =2.7 ± 0.8; umbilical port VAS score= 2.04 ± 0.7), at the 24-hour mark (epigastric port VAS scores =2.1 ± 0.7; umbilical port VAS scores 1.17 ± 0.6). Finally, during the 36-hour post-surgery period, the epigastric port still had higher VAS scores (1.3 ± 0.7) compared to the umbilical port (0.9 ± 0.4), and all the differences at different time intervals were found to be statistically significant (p<0.05). Additionally, we found that retrieval difficulty, as indicated by the retrieval difficulty score, was statistically significantly higher in the epigastric port (4.4 ± 1.2) compared to the umbilical port (3.9 ± 1.1; p=0.01) (Table 3).

**Table 3:** Comparison of pain score, visual analog scale (VAS) scores, and retrieval difficulty between the umbilical and the epigastric port in laparoscopic cholecystectomy patients. Significant = p<0.05

	Epigastric port (n=25)	Umbilical port (n=25)	p-value
VAS score			
1 hour	6.9 ± 1.2	5.1 ± 1.5	0.001
6 hours	4.9 ± 0.4	2.5 ± 1.5	0.03
12 hours	2.7 ± 0.8	2.04 ± 0.7	0.01
24 hours	2.1 ± 0.7	1.17 ± 0.6	0.02
36 hours	1.3 ± 0.7	0.9 ± 0.4	0.01
Retrieval difficulty	4.4 ± 1.2	3.9 ± 1.1	0.01

## Discussion

This prospective randomized controlled trial aims to appraise the occurrence of postoperative PSI following LC via either the umbilical or epigastric port. Through meticulous methodology and comprehensive analysis, we attempt to highlight the crucial facet of postoperative care that potentially influences improving surgical protocols and patient management strategies. Based on our study results, we reject the null hypothesis, indicating that LC conducted via the umbilical port carries a lower risk of developing PSI compared to the epigastric port. Our study observed a predominance of female patients (61%) undergoing LC, aligning with previous investigations by Siddiqui et al., Shakya et al., Bashir et al., and Ahmad et al. [7-10]. This observation can be attributed to the prevalence of benign GB diseases, such as GB stones or polyps, among females, which is approximately 2-3 times more common than males [12]. Factors contributing to this increased risk in females include post-pregnancy changes, contraceptive use, estrogen-related effects, and hormonal replacement therapy [13,14].

The study participants’ mean age was 43.5 ± 10.7 for the epigastric port and 40.7 ± 12.6 for the umbilical port, consistent with findings from Siddiqui et al. (42.5 ± 10.7 vs. 40.6 ± 12.6) and Bashir et al. (47.49 ± 9.4 vs. 46.8 ± 5.6) [7,9]. A significant proportion of patients (66%) in our study presented with symptomatic cholelithiasis, corroborating previous reports, indicating GB stones as the primary indication for LC [7]. Postoperative PSI in LC typically arises from port site infection by endogenous flora or contamination with infected bile [15]. Our study revealed an overall PSI incidence of 12% (6/50 patients), which was higher than reported in existing studies by Mir et al. (6.7%), Saud and AbuAl-Hail (2.4%), and Al-Naser (4.5%) [16-18]. Discrepancies among these findings may be attributed to population diversity, variations in patient immune responses, hospital environments, ergonomics, and equipment sterilization.

When PSI was assessed at POD 10 and POD 30, the umbilical port site exhibited a non-significantly higher infection rate compared to the epigastric port site. A similar higher PSI was found in the findings of Sainia et al. and Chopra et al. [19-20]. However, Al-Naser reported higher PSI rates in the epigastric port site [18]. The reason for the higher PSI rate at the umbilical port site might be the potentially elevated microbial load that persists even after antiseptic cleaning. Additionally, port site scarring was cosmetically more favorable at the umbilical port site, aligning with the results of Sainia et al., possibly due to the umbilical creases concealing the scar [19]. Our study observed that GB retrieval through the umbilical port resulted in a relatively lower mean retrieval difficulty score than the epigastric port, with a difference of 0.5. This finding contradicts studies by Siddiqui et al. and Hajong et al. but is consistent with Ahmad et al. and Bashir et al. [7,9,10,21]. Variations among these studies may arise from population differences, incision length, GB retrieval techniques, and surgeons’ subjective perceptions of difficulty scores [7,21]. The sheath’s long 5 mm stab incision facilitates a wider opening for GB retrieval.

Regarding the intensity of POP assessed using the VAS, patients in the epigastric port reported statistically significant higher pain scores than those in the umbilical port at different time intervals. These findings were in accordance with the study conducted by Siddiqui et al., Sainia et al., and Hajong et al. [7,20,21]. However, on the contrary, Bashir et al. and Ahmad et al. presented equal effectiveness of both ports regarding POP [9,10]. This implies that the choice of GB retrieval site may influence the severity of postoperative PSI. The exact mechanism underlying these differences remains unclear. Still, it may be related to the higher concentration of nerve endings in the epigastric region, rendering it more pain-sensitive than the umbilical region.

These findings hold significance for surgical practice, suggesting that the site of GB retrieval can impact the severity of postoperative PSI and pain. However, our study faced some inherent limitations such as a small sample size, short follow-up duration, and a single-center setting. Pain perception is subjective and influenced by various factors, such as individual pain tolerance and analgesic use. Therefore, we recommend further high-quality, multicenter, longitudinal, randomized controlled trials to validate these findings and explore additional strategies for minimizing postoperative infection and pain following LC.

## Conclusions

In our comparative study of LC via epigastric and umbilical ports, we observed favorable outcomes associated with the umbilical port, including a reduced risk of postoperative PSI. On the other hand, the epigastric port demonstrated a higher likelihood of PSI occurrence. Additionally, using the umbilical port was a better alternative for experiencing less POP, achieving a more aesthetically pleasing port site scar appearance, and encountering lower GB retrieval difficulty. These findings suggest that the port site choice can significantly influence clinical and patient-reported outcomes in LC. Our study contributes valuable insights into minimally invasive surgery and patient care, however, further research is warranted to validate and generalize these results.
